# Intra-operative fiducial-based CT/fluoroscope image registration framework for image-guided robot-assisted joint fracture surgery

**DOI:** 10.1007/s11548-017-1602-9

**Published:** 2017-05-04

**Authors:** Giulio Dagnino, Ioannis Georgilas, Samir Morad, Peter Gibbons, Payam Tarassoli, Roger Atkins, Sanja Dogramadzi

**Affiliations:** 1 Bristol Robotics Laboratory, University of the West of England, Coldharbour Lane, BS161QY Bristol, UK; 2 Aston University, B47ET Birmingham, UK; 3 University Hospitals Bristol, Upper Maudlin Street, BS28HW Bristol, UK

**Keywords:** Intra-operative registration, Image-guided surgery, Minimally invasive fracture surgery, Robot-assisted surgery, CT/fluoroscopy data

## Abstract

**Purpose:**

Joint fractures must be accurately reduced minimising soft tissue damages to avoid negative surgical outcomes. To this regard, we have developed the RAFS surgical system, which allows the percutaneous reduction of intra-articular fractures and provides intra-operative real-time 3D image guidance to the surgeon. Earlier experiments showed the effectiveness of the RAFS system on phantoms, but also key issues which precluded its use in a clinical application. This work proposes a redesign of the RAFS’s navigation system overcoming the earlier version’s issues, aiming to move the RAFS system into a surgical environment.

**Methods:**

The navigation system is improved through an image registration framework allowing the intra-operative registration between pre-operative CT images and intra-operative fluoroscopic images of a fractured bone using a custom-made fiducial marker. The objective of the registration is to estimate the relative pose between a bone fragment and an orthopaedic manipulation pin inserted into it intra-operatively. The actual pose of the bone fragment can be updated in real time using an optical tracker, enabling the image guidance.

**Results:**

Experiments on phantom and cadavers demonstrated the accuracy and reliability of the registration framework, showing a reduction accuracy (sTRE) of about $0.88~\pm 0.2\,\hbox {mm}$ (phantom) and $1.15\pm 0.8\,\hbox {mm}$ (cadavers). Four distal femur fractures were successfully reduced in cadaveric specimens using the improved navigation system and the RAFS system following the new clinical workflow (reduction error $1.2\pm 0.3\,\hbox {mm}$, $2\pm 1{^{\circ }})$.

**Conclusion:**

Experiments showed the feasibility of the image registration framework. It was successfully integrated into the navigation system, allowing the use of the RAFS system in a realistic surgical application.

## Introduction

The goal of fracture surgery is to get the bone to heal by accurately aligning and fixing the broken fragments [1]. Minimally invasive procedures aim to minimise the soft tissue damage by manipulating the fragments through small incisions, reducing also the risk of infections and allowing a quicker recovery time [2]. However, these techniques are limited by the surgeons’ ability to achieve accurate fracture reduction using 2D intra-operative fluoroscopic imaging to solve the 3D fragment alignment. This can be difficult to achieve, as 2D fluoroscopic images lack 3D spatial information making accurate reduction planning and evaluation difficult [3]. This is even more difficult for joint fractures which present a 3D problem requiring restoring three translations and three rotations to achieve optimal reconstruction of the articular surface [4]. Moreover, the procedure necessitates repeated images being taken which increase radiation exposure to patient and staff [5] or, on occasions, expensive revision operations to correct malposition [6].

Intra-operative image guidance can potentially have a positive impact in overcoming the issues identified above through enhanced 3D imaging and increased reduction accuracy [7]. Image registration is one of the key factors affecting the accuracy of image-guided surgical systems as it maps the pre-operative information into surgical reality [8], bringing pre-operative images (e.g. models of patient anatomy) and intra-operative images (e.g. patient’s images, pose of tools) into the same coordinate frame, and providing the surgeon with a better image guidance [3]. In fracture surgery, pre-operative images are usually 3D data provided by CT scans (i.e. CT-generated 3D model of the fracture), while intra-operative images can be either 2D data provided by a fluoroscope (i.e. the fracture), or 3D digitalised surfaces (i.e. CAD models of surgical tools). Therefore, image registration can be either 2D/3D or 3D/3D [3]. Accurate image registration between pre- and intra-operative images allows not only precise intra-operative navigation but also the possibility of pre-planning the surgical procedure on CT images and mapping the pre-defined surgical paths intra-operatively [9].

Intra-operative surgical guidance for orthopaedic surgery implant positioning is found in the commercially available systems of Stryker (e.g. OrthoMap), Smith & Nephew (e.g. Trigen) and Brainlab (e.g. Knee3). Several surgical systems which integrate robotic assistance and 3D image guidance are reported in the literature [10–16]. However, all the above systems deal with long bone fractures and they have not been designed to be used for joint fracture surgery image guidance, as this typically requires higher reduction accuracy to restore the articular surface [17]. To the best of authors’ knowledge, no image guidance system for joint fracture reduction has been reported in the literature. Earlier research on minimally invasive joint fracture surgery by the authors of this paper resulted in an image-guided robotic system (i.e. the robot-assisted fracture surgery (RAFS) system) that can successfully accomplish the reduction of 1-fragment distal femur fracture on phantom models [7]. However, within the ultimate aim to move the system into a clinical scenario, key issues (described below) related to the navigation system and the clinical workflow were identified. This paper proposes a clinically usable bespoke framework for intra-operative fiducial-based image registration of pre-operative CT images and intra-operative fluoroscopic images, enabling image guidance for the RAFS system and allowing its integration in a real surgical environment. The main technical contributions of this study are: (1) fiducial-based pose estimation of fluoroscopic images; (2) intra-operative CT/fluoroscopic image registration; and (3) experimental validation using a plastic phantom and cadaver specimens in a realistic surgical scenario. We demonstrate the performance of the proposed framework within the context of image-guided robot-assisted distal femur fracture surgery, where image registration is used to estimate the relative pose of bone fragments and inserted surgical tools manipulated by our robotic system.

## Background

RAFS is an image-guided robotic system designed to reduce intra-articular fractures percutaneously. The key aspects of the RAFS system are: improved reduction accuracy, minimised soft tissue damage, full pre-operative planning, and enhanced intra-operative real-time 3D image guidance [7, 19]. The overall architecture of the RAFS system is based on a host–target configuration (see [20]), with the surgical team always in control of the entire system. The surgeon pre-plans the surgical procedure from the system workstation by virtually reducing the fracture. The high-level controller processes the surgeon’s commands and generates the motion commands for the robot to achieve the planned reduction. Position control of the robot is based on closed-loop position controllers [21]. External position measurements are necessary for the overall system accuracy and repeatability: visual feedback is gained by optical tracking data in order to implement closed-loop vision-based control on the robot by placing an optical tool on each end-effector. Force/torque feedback is gained by a 6-DOF load cell mounted on each robot’s end-effector. These feedback data are used as a safety feature for the system: if the measured force/torque data exceed pre-defined safety thresholds (measured in [22, 23]), then the force controller immediately stops the movement of the robot to avoid damages to the patient. The control architecture for the RAFS system is fully reported in [18].

**Fig. 1:**
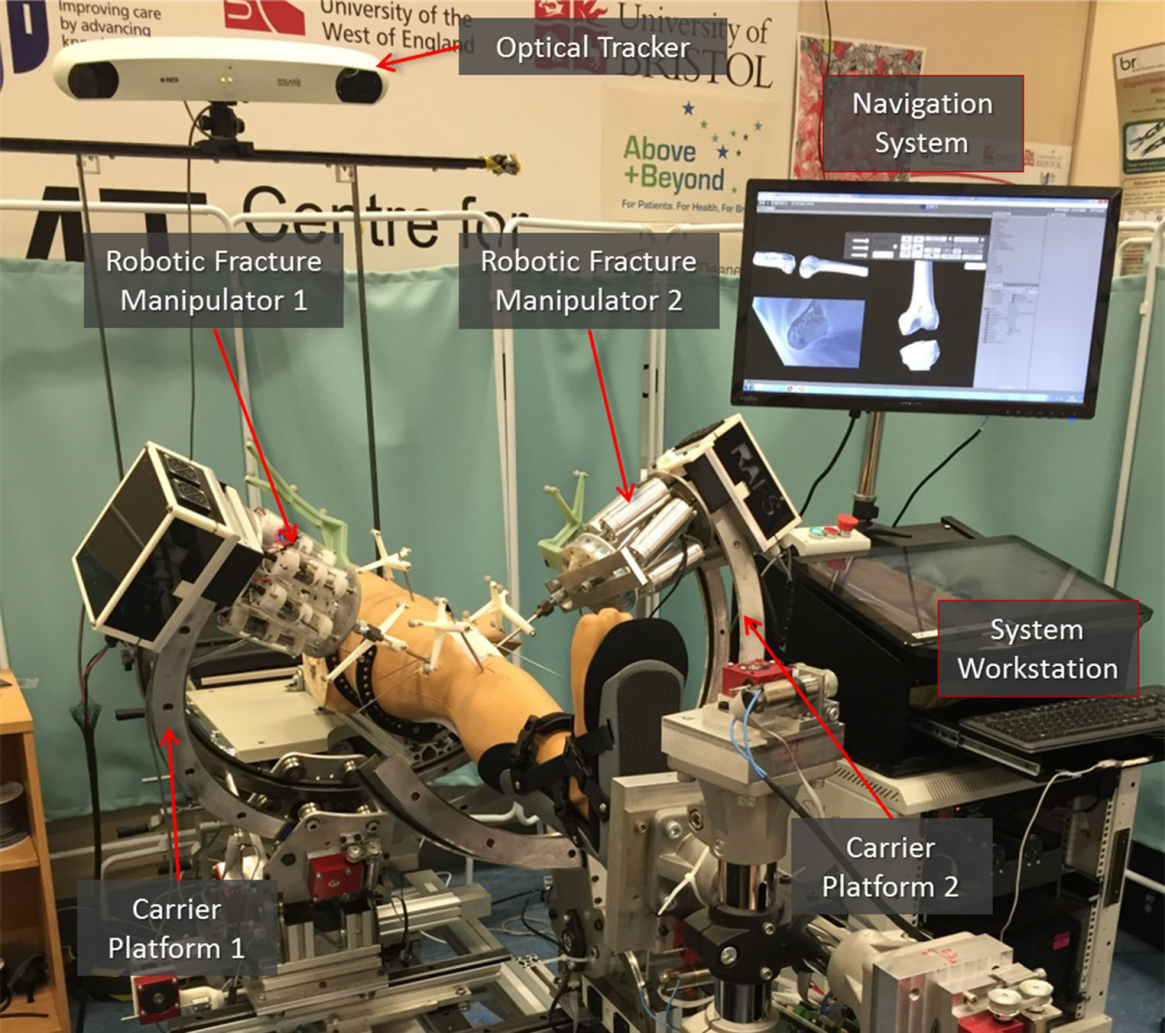
The RAFS surgical system. Two robotic fracture manipulators, mounted on two carrier platforms, are percutaneously connected to a knee fracture. The robotic system is operated by the surgeon through the system workstation. The navigation system provides the surgeon with intra-operative 3D image guidance by updating in real time the pose of the bone fragments using an optical tracker

This research improves the earlier RAFS surgical system prototype described in [18] and its navigation system described in [7, 19]. The new RAFS system prototype, shown in Fig. 1, is briefly described below.

### RAFS surgical system

The expectations for the RAFS surgical system can be summarised in 4 points: (1) reduction of complex joint fractures by manipulating two bone fragments simultaneously to precisely align them and restore the joint functionality; (2) minimisation of the soft tissue damage by percutaneous manipulation of the bone fragments; (3) full pre-operative planning of the reduction procedure and enhanced intra-operative 3D image guidance; (4) integration and testing in a realistic surgical environment. The RAFS system consists of the following components:

*Two robotic fracture manipulators (RFMs) and two carrier platforms (CPs):* The RFM, introduced in [20], is a computer-controlled robotic device used to manipulate a bone fragment through an orthopaedic pin connected to it ($0.03\pm 0.01$ mm translational accuracy and $0.12\pm 0.01{^{\circ }}$ rotational accuracy [20]). Each RFM is mounted on a bespoke CP (4 DOFs, computer controlled) which is used for the coarse positioning of the RFM close to the manipulation pin as described in [18]. The implementation of two RFM–CP systems allows the simultaneous manipulation of two bone fragments. The kinematics and control of the RFM and the CP are described in [18].

*System workstation:* The graphical user interface (GUI) allows the surgeon to interact with the RFMs and CPs through the navigation system. The high-level control—running on a real-time controller with FPGA (NI compactRIO 9068, National Instruments)—processes the user’s commands and generates the motion commands for the low-level controller (EPOS 2 24/3, Maxon Motor) which controls the movement of the robotic system [18].

*Navigation system:* It consists of a reduction software, an optical tracking system, and a user controller. The navigation system allows the surgeon to fully pre-plan the surgical procedure by virtually reducing the fracture, i.e. manipulating 3D models of the broken bones generated by the pre-operative CT data using the user controller [19]. The optical tracker (Polaris Spectra, NDI Inc.) enables the intra-operative image guidance by updating in real time (25Hz) the pose of the 3D models of the bones during the surgery, through optical tools placed on the orthopaedic pins inserted into the bones (Fig. 1). The navigation system architecture is described in [7].

**Fig. 2:**
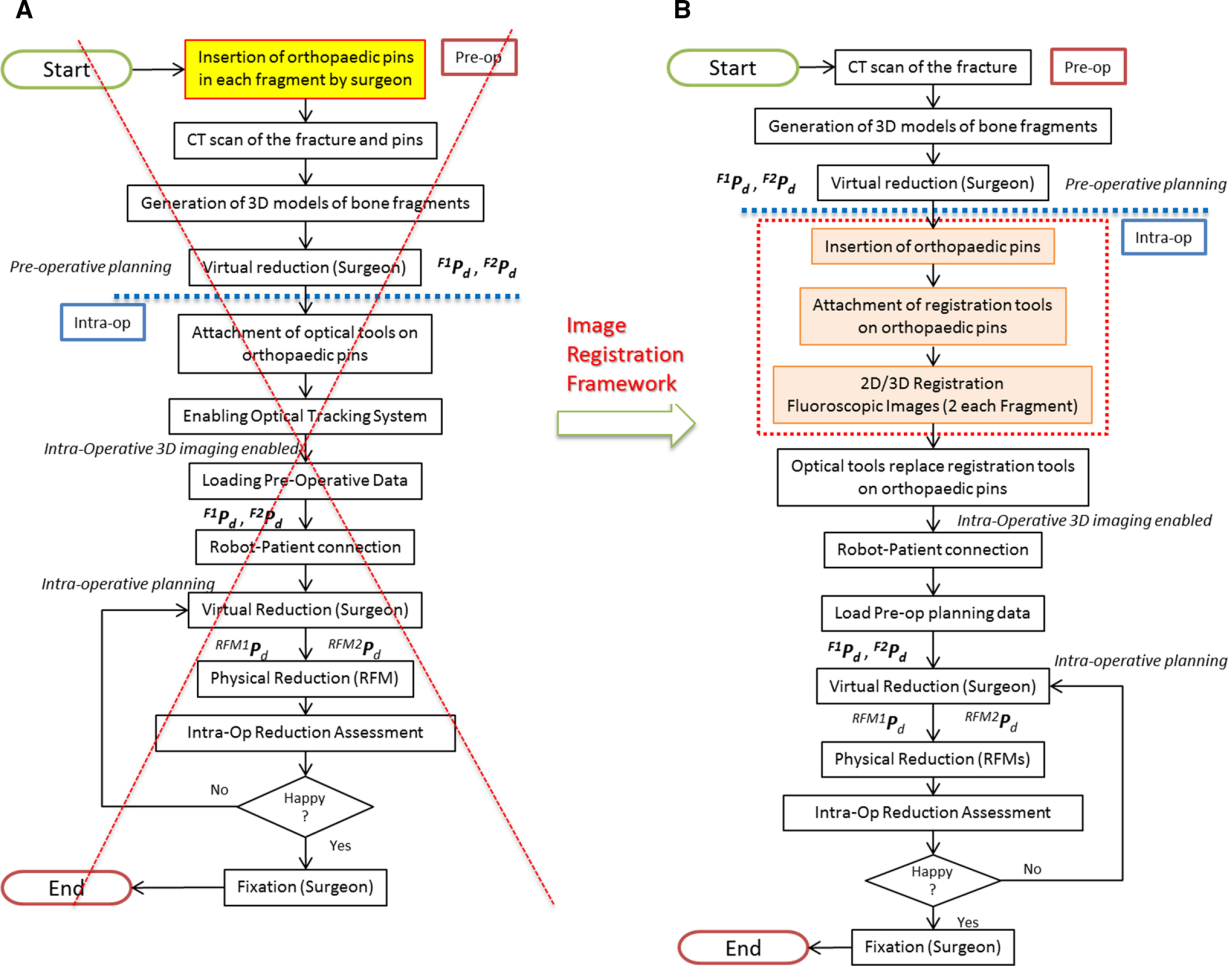
RAFS clinical workflow. The old clinical workflow (**a**) has been modified allowing the RAFS system to be used in a real surgical procedure. The new clinical workflow is shown in **b**

### Limitations of the earlier navigation system

Experiments with the earlier navigation system used in combination with the previous prototype of the robotic system on bone plastic models resulted in a fracture reduction accuracy of about 1 mm and $1.5{^{\circ }}$ as reported in [7]. However, with the ultimate aim to move the system into a realistic clinical scenario, a key issue was identified. The pre-operative part of the clinical workflow for the reduction of joint fractures using the earlier navigation system (Fig. 2a, refer to [7]) starts with the insertion of the orthopaedic pins in the fragments, followed by the CT scan of the fracture with pins inserted and the creation of the corresponding 3D models. Since each pin is rigidly connected to the fragment, the actual pose of the fragment can be updated by the optical tracker by placing an optical tool on top of the pin, enabling the intra-operative 3D image guidance. This workflow presents one main issue, i.e. the pins are inserted into the bones *before* getting the CT scan. This should be done in the operating theatre as it is a surgical procedure requiring an adequate aseptic environment and the patient undergoing a general anaesthetic. Having a CT scanner or a 3D fluoroscopic imager in the operating theatre could be a solution, but this is generally not the case in most hospitals because of the high costs involved [24]. Realistically, the pins should be inserted into the fragments *after* getting the CT scan of the fracture. Moreover, this would allow the full pre-operative planning, as the surgeon can accurately plan the procedure just after getting the CT scan and *before* the surgery starts, thus potentially reducing the surgical time. However, this requires a new clinical workflow and redesigned navigation system.

## New clinical workflow

**Fig. 3:**
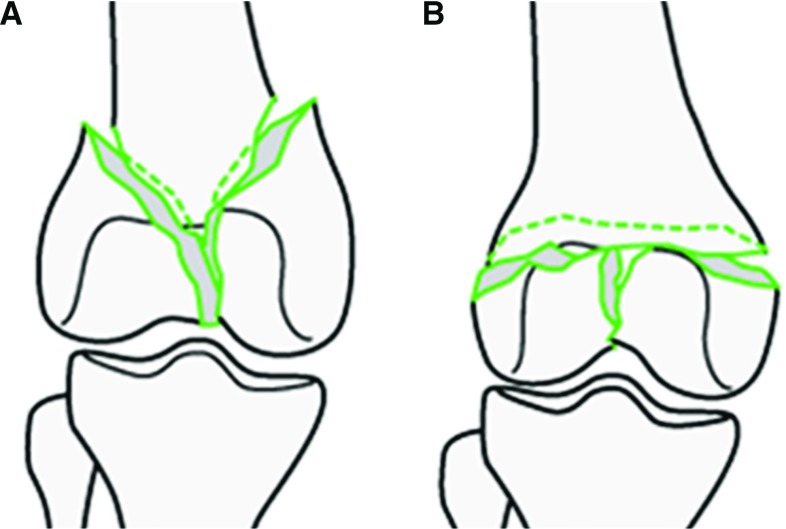
Complete articular distal femur fractures: Y-shaped 33-C1 (**a**) and T-shaped 33-C1 (**b**) used in this study. Picture from [25]

This section describes the new clinical workflow for the reduction of joint fractures using the RAFS surgical system in a realistic surgical environment. Figure 2 shows the earlier clinical workflow (Fig. 2a) [7] and the new one (Fig. 2b). Complex three-part distal femur fractures (DFFs) such as Y- and T-shaped 33-C1 (Fig. 3) [25] have been used in this study.

**Fig. 4:**
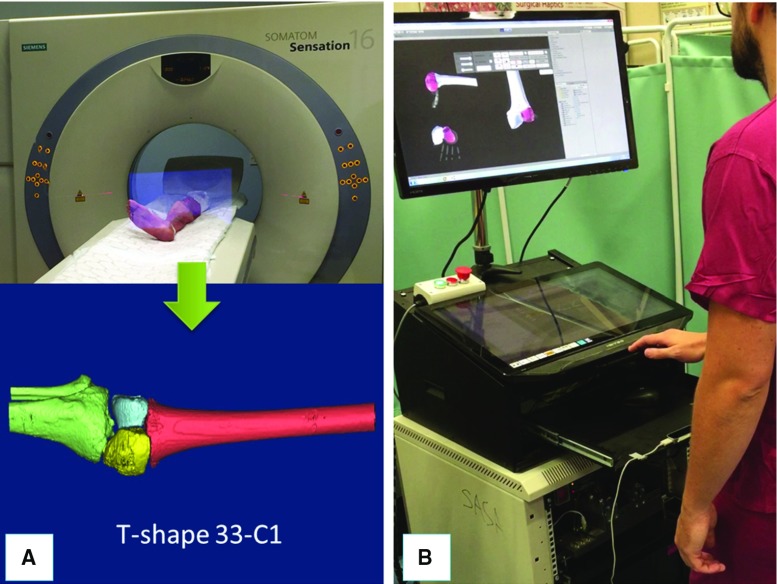
Pre-operative operations. Example of 3D model of a T-shaped 33-C1 distal femur fracture generated by CT scanning a cadaveric specimen (**a**); a surgeon virtually reducing the fracture using the navigation system (**b**)

### Pre-operative procedure

The procedure starts with a pre-operative CT scan of the fracture. The resulting dataset is segmented using commercial software (ImageSim, Volmo Ltd, Newbury, UK) to generate 3D models (STL format) of each bone fragment. The surgeon virtually reduces the fracture using the reduction software GUI, i.e. manipulates the fragments F1 and F2 to match the femur FEM (Fig. 4). The pose of F1 and F2 with respect to FEM in the desired (*d*) reduced configuration ($^{{\textit{F1}}}{\varvec{P}}_{d}$, $^{{\textit{F2}}}{\varvec{P}}_{d})$ is stored in the system, concluding the pre-operative part of the procedure. Pre-operative data are then used intra-operatively to achieve the physical reduction of the fracture using the robotic system [7].

**Fig. 5:**
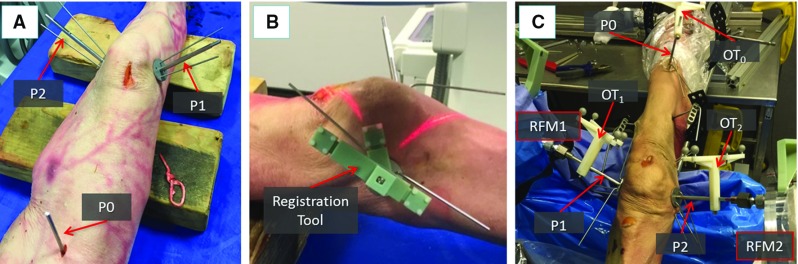
Intra-operative operations. Orthopaedic pins inserted into the bone fragments of a cadaveric specimen (**a**). Example of a registration tool placed on orthopaedic pin for the registration procedure (**b**). Optical tools replace registration tools on top of each pin, enabling the intra-operative image guidance. RFMs are then connected to the manipulation pins to reduce the fracture (**c**)

**Fig. 6:**
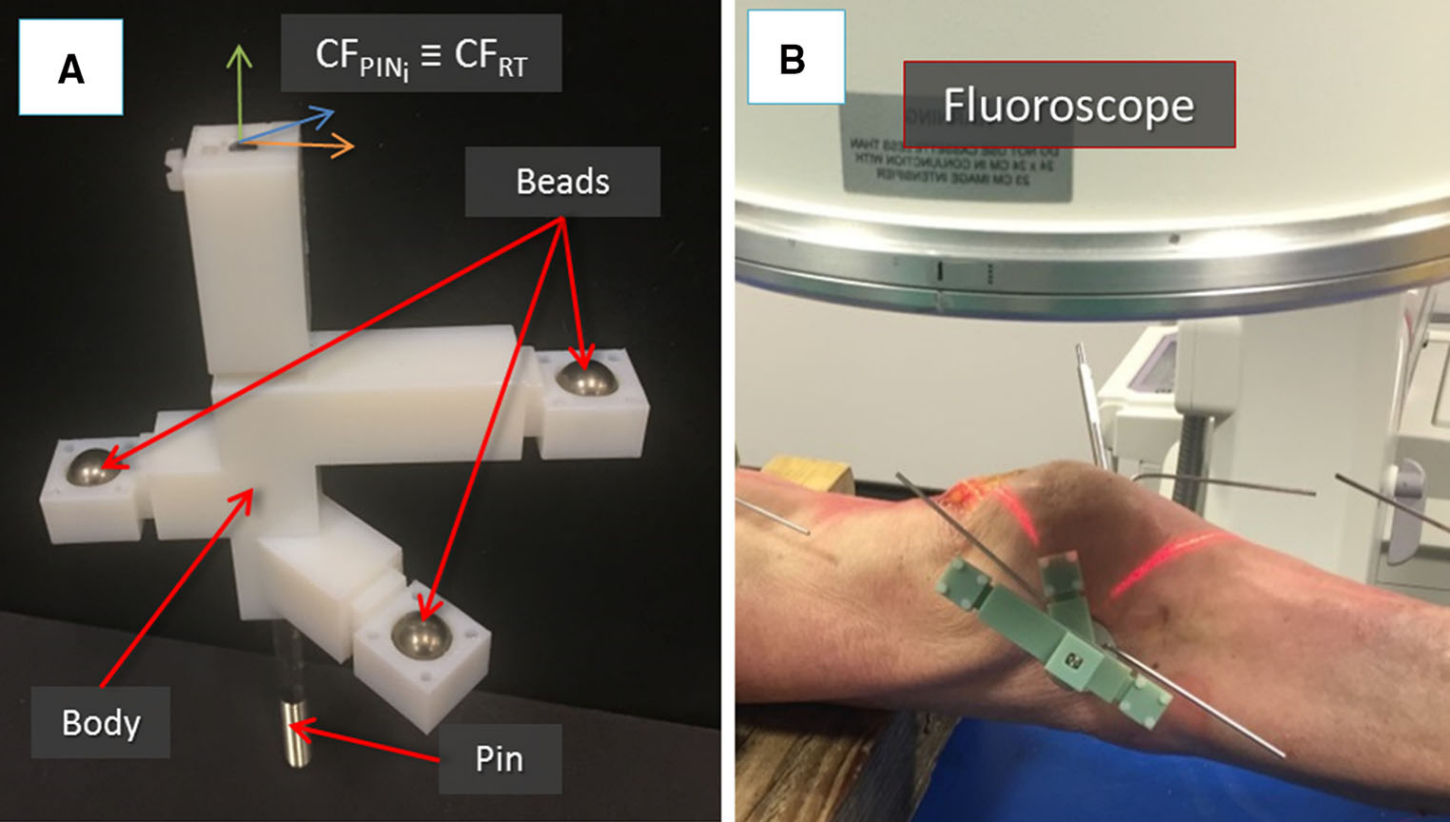
Image registration framework: the registration tool used for the pose estimation of fluoroscopic images. The pin and the registration tool have coincident coordinate frames (**a**). The registration tool placed on a pin inserted into a fractured bone fragment of a cadaveric specimen ready for fluoroscopic imaging (**b**)

### Intra-operative procedure

Patient is moved to the operating theatre and undergoes general anaesthesia. Three orthopaedic pins are inserted into the bone fragments (Fig. 5a) through small incision in the flash, minimising the soft tissue damage. Namely, P1 is inserted in F1, P2 in F2, and P0 in FEM. In order to enable intra-operative image guidance—which allows the surgeon to navigate the fracture and achieve the robotic reduction—the relative position of each pin with respect to the bone fragment in which it is inserted needs to be calculated. This is achieved through an intra-operative fiducial-based image registration framework. To this regard, a custom-made fiducial marker, i.e. the registration tool (RT), has been designed (see Figs. 5b, 6). A RT is placed on each orthopaedic pin (an example is shown in Fig. 5b), and the registration framework executed as described in the next section. Once the registration is completed, the relative pose between each pin and its fragment is known, and the homogeneous transformations $^{{\textit{Pi}}}{\varvec{T}}_{{\textit{Fi}}}$ can be calculated (see Fig. 8). Registration tools are then removed from the orthopaedic pins and replaced by the optical tools: $\hbox {OT}_{1}$ on P1, $\hbox {OT}_{2}$ on P2, and $\hbox {OT}_{0}$ on P0 (Fig. 5c). Orthopaedic pins and optical tools were designed in a unique way to have their coordinate frames coincident, i.e. $\hbox {CF}_{{\mathrm{PINi}}}\equiv \hbox {CF}_{{\mathrm{OTi}}}$ (see [7]). Therefore, the optical tracker provides the actual (*a*) pose of each bone ($^{{\textit{F1}}}{\varvec{P}}_{a}$, $^{{\textit{F2}}}{\varvec{P}}_{a}$, $^{{\textit{FEM}}}{\varvec{P}}_{a})$ by tracking its pin. This establishes a direct correspondence between the image space (reduction software, virtual models) and the task space (real fracture) described by $^{{\textit{IMG}}}{\varvec{T}}_{{\textit{Fi}}}$. A full description of the coordinate frames and transformations involved is provided in [7]. Once the intra-operative real-time image guidance is enabled, the robotic system is connected to the patient: the CPs move the RFMs close to the manipulation pins P1 and P2 and the surgeon’s assistant connects RFM1 to P1, and RFM2 to P2 (Fig. 5c). Results of the pre-operative planning ($^{{\textit{F1}}}{\varvec{P}}_{d}$, $^{{\textit{F2}}}{\varvec{P}}_{d}$) are uploaded into the intra-operative procedure, and the corresponding desired poses in the task space for the RFMs $^{{\text {RFM1}}}{\varvec{P}}_{d}$ and $^{{\text {RFM2}}}{\varvec{P}}_{d}$ are calculated to achieve the fracture reduction as (see [7]):

1 $$\begin{aligned} {}^{{\text {RFM}}1}{\varvec{P}}_{d} = {}^{{\text {RFM}}1}{\varvec{T}}_{P1} \times {}^{{\text {IMG}}}T_{{{\mathrm{F}}}1} \times {}^{F1}{\varvec{P}}_{d} \nonumber \\ {}^{{\text {RFM}}2}{\varvec{P}}_{d} ={}^{{\text {RFM}}2}{\varvec{T}}_{P2} \times {}^{{\text {IMG}}}T_{{\mathrm{F}}2} \times {}^{F2}{\varvec{P}}_{d} \end{aligned}$$

where $^{{\text {RFM1}}}{\varvec{T}}_{P1}$ and $^{{\text {RFM2}}}{\varvec{T}}_{P2}$ are the homogeneous transformations between the RFM1 and P1, and RFM2 and P2, respectively. These are provided by the optical tracker by tracking two optical tools placed on the RFMs [7].

The RFMs automatically move F1 and F2 to achieve the physical reduction of the fracture (FEM remains static), based on the virtual reduction, i.e. the pre-operative planning data ($^{{\textit{F1}}}{\varvec{P}}_{d}$, $^{{\textit{F2}}}{\varvec{P}}_{d}$), performed by the surgeon [7]. The navigation system provides the surgeon with the actual pose of the fragments in real time, so that he/she can assess the reduction in 3D intra-operatively without the use of X-ray-based imaging devices. The surgeon is always in control of the system and can take over at any time by modifying the reduction paths performing an intra-operative virtual reduction to move the RFMs along new desired trajectories. As this is not a teleoperated system, the RFMs do not move during the intra-operative virtual reduction. They are reactivated by the surgeon once the new trajectories are redefined. Finally, once the surgeon is happy with the physical reduction, he/she fixates the fracture, and the surgery ends.

## Navigation system: image registration framework

In order to update the new clinical workflow, i.e. to calculate the relative pose between each pin and its fragment intra-operatively ($^{{\textit{Pi}}}{\varvec{T}}_{{\textit{Fi}}})$, the navigation system introduced in [7] has been redesigned as follows. The reduction software, as in the earlier version, receives pre-operative data from the CT scanner and generates and displays 3D models of the bone fragments which can be manipulated by the surgeon to achieve the virtual reduction. Also, the reduction software now receives intra-operative fluoroscopic images of the fractured fragments and inserted pins. The registration framework registers them with the pre-operative CT dataset through a fiducial marker of known geometry placed on the pins, calculating the homogeneous transformations $^{{\textit{Pi}}}{\varvec{T}}_{{\textit{Fi}}}$.

### Problem definition

Given two projection images (fluoroscopic images) of a bone fragment and inserted pin with a fiducial marker of known geometry, we aim to compute the relative pose between (1) the images and the fiducial, and (2) the bone fragment and the images. This defines the relative pose of each pin with respect to the fragment (i.e. the fragment in which the pin is inserted), enabling the intra-operative 3D image guidance.

### Pre-operative procedures: C-arm fluoroscope calibration

In order to achieve a precise image registration, it is essential to define a proper model and imaging geometry of the C-arm fluoroscope, and correct the image distortion. Therefore, the C-arm intrinsic parameters (i.e. focal length, pixel spacing) should be calculated [26]. In this study, we used an OEC Fluorostar (GE, Salt Lake City, UT) C-arm fluoroscope with a 230-mm image intensifier and a source-to-image distance (SID) of 980 mm. For this C-arm system, the SID is constant and denotes the focal length. Moreover, considering that the C-arm detector is not a flat panel, image distortion correction is required [27]. The distortion is mainly due to the mapping of the planar image on the curved input phosphor of the image intensifier which results in a stretching of the image. As the distortion is radial dependent, it is most apparent at the periphery of the field of view. Several approaches to correct this distortion are reported in the literature [28]. We used a custom-designed calibration phantom composed by a grid of 10-mm metallic ball bearings uniformly distributed on a plane with a known, constant displacement in the *x* and *y* directions ($d_{x} = d_{y} =22$ mm). The calibration phantom was placed on the C-arm detector, and one fluoroscopic image was acquired. The grid was segmented in the image, and a global method of distortion correction using two-dimensional polynomial was applied to the segmented beads to estimate the distortion pattern and calibrate the image [29]. The acquired image was also used to estimate the pixel size (PS) of the image intensifier which was found to be 0.224 mm, completing the pre-operative definition of the required C-arm intrinsic parameters.

### Registration framework

The proposed registration framework involves: (1) the estimation of relative pose between two fluoroscopic images using a custom-made fiducial marker; (2) registration between the 3D model of bone fragments and the fluoroscopic images. The registration workflow is described below.

**Fig. 7:**
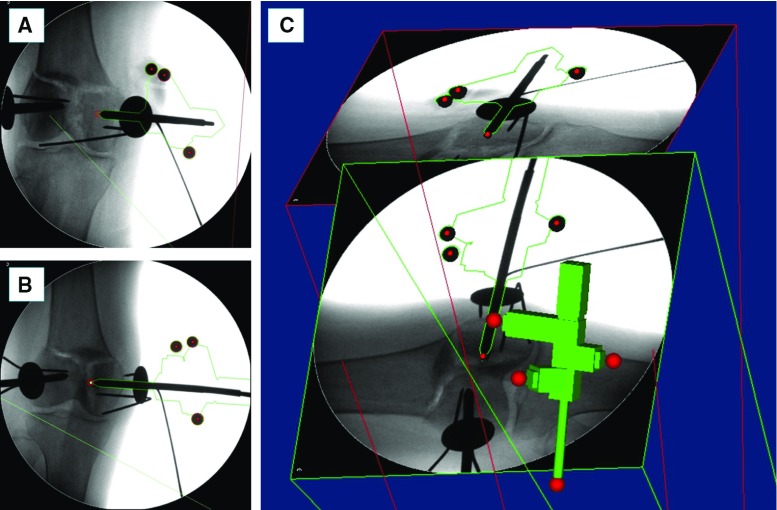
Image registration framework: fluoroscopic images pose estimation. Two fluoroscopic images of the registration tool, pin, and fractured bone (**a**, **b**) are imported into the reduction software together with the CAD 3D model of the registration tool and the pin (**c**). Pose of the fluoroscopic images is estimated using point correspondence between the known features in the CAD 3D model (*red spheres* in **c**) and in the images (*red circles* in **a** and **b**)

**Fig. 8:**
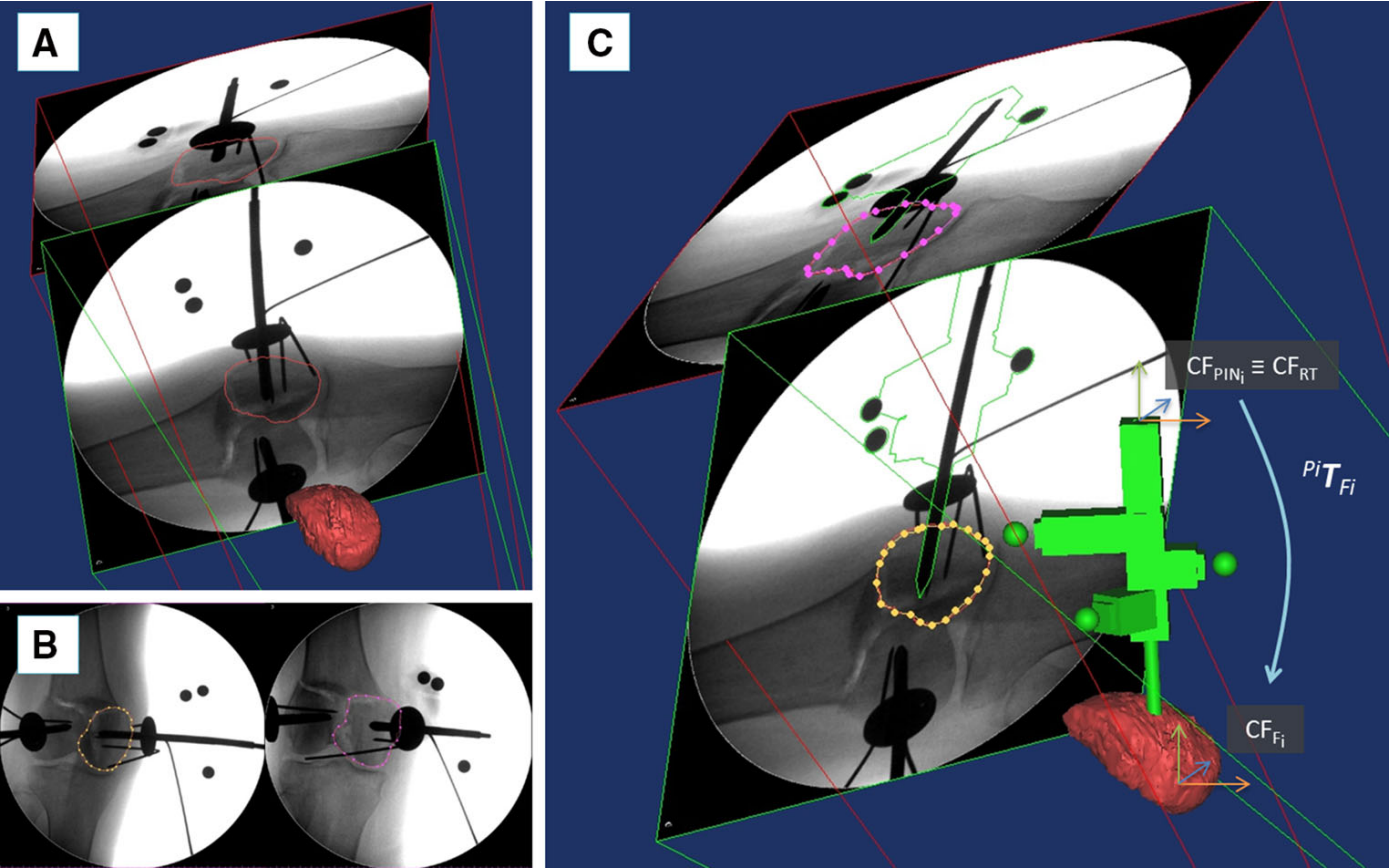
Image registration framework: registration. The CT-generated 3D model of a fractured bone fragment is imported into the reduction software and its contour projected onto the two fluoroscopic images (**a**). The contour of the corresponding bone fragment is segmented on the fluoroscopic images (**b**). The 3D model of the bone is registered with the fluoroscopic images, and the relative pose of the pin with respect to the bone is calculated (**c**)

1. *Fluoroscopic images pose estimation: *The custom-made fiducial marker, i.e. the registration tool (RT), used for the pose estimation of the fluoroscopic images is shown in Fig. 6. The RT contains three stainless steel beads (radiopaque) visible in fluoroscopic images. The beads are encapsulated into the body of the RT made of acrylonitrile-butadiene-styrene (ADP), which is a radiolucent material not visible in fluoroscopic images (Fig. 6a). The RT is placed on the orthopaedic pin (previously inserted into the bone fragment) as shown in Figs. 5b, 6b, and two fluoroscopic images of the RT+pin+bone are taken from two different views, i.e. anterior–posterior (AP, e.g. $90{^{\circ }}$) and lateral (LAT, e.g. $60{^{\circ }}$). The RT has been designed to be rigidly connected in a unique way to the orthopaedic pin, having their coordinate frame coincident, i.e. $\hbox {CF}_{{\mathrm{PINi}}}\equiv \hbox {CF}_{{\mathrm{RT}}}$ (Fig. 6a); therefore, the pose of the pin can be estimated through the RT. The images are imported into the reduction software together with the 3D model of the RT and the orthopaedic pin (i.e. its CAD model) (Fig. 7). The 6-DOF pose of the images is estimated using the projection of the known feature geometries of the RT (beads) and the pin (sharp bottom end). The location of these features is manually identified in the fluoroscopic images by using filtering and thresholding (Fig. 7a, b) [30]. A registration algorithm [31] is used to estimate the 6-DOF pose of the fluoroscopic images with respect to the RT $+$ pin using point correspondence between the known features in the CAD 3D model and in the images (Fig. 7c).
2. *Registration: *Once the relative pose of the fluoroscopic images has been calculated, the pose of the fractured bone fragment should be estimated (Fig. 8). The 3D model of the bone fragment (pre-operatively generated by the CT data as described in the previous section) is imported into the reduction software, and a projection contour of the 3D model of the bone is generated (Fig. 8a). The surgeon manually translates and rotates the 3D model of the bone to match its projected contour with the corresponding contour of the fragment in both 2D fluoroscopic images. The result of this coarse registration is the input to the next optimisation step. The contour of the bone fragment is segmented on each fluoroscopic image (Fig. 8b) using the technique proposed by Guéziec et al. [32]. A spline-based registration method [33] is applied to minimise the distance between the projected 3D contour of the model and the segmented 2D contour on each fluoroscopic image (Fig. 8c). The 3D model of the bone is now registered with the fluoroscopic images, and its relative pose with respect to the pin is known and described by the homogeneous transformations $^{{\textit{Pi}}}{\varvec{T}}_{{\textit{Fi}}}$ (Fig. 8c). $^{{\textit{Pi}}}{\varvec{T}}_{{\textit{Fi}}}$ is considered to be constant during the operation. As a result, the pose of the bone fragment can be updated in real time by connecting the optical tool to the pin, as described in the clinical workflow.

## Experimental validation

The proposed registration framework has been tested through two sets of experiments on complex intra-articular distal femur fractures (33-C1, Fig. 3). The framework was initially tested in laboratory on a plastic phantom for initial performance evaluation. It was assessed on 13 cadaveric specimens to verify its accuracy in a realistic surgical environment. Also, 4 fractures were reduced using the new navigation system and the RAFS surgical system. The pre-operative CT images were acquired with a SOMATOM Sensation 16 (Siemens Healthcare, Erlangen, Germany) CT scanner, with a voxel size of $0.58\,\hbox {mm}\times 0.58\,\hbox {mm}\times 0.75\,\hbox {mm}$. Intra-operative fluoroscopic images were acquired with the OEC Fluorostar C-arm fluoroscope; we calibrated the C-arm prior to the experiments, and the calibration parameters were used for all the acquired images for registration.

### Evaluation methodology

**Fig. 9:**
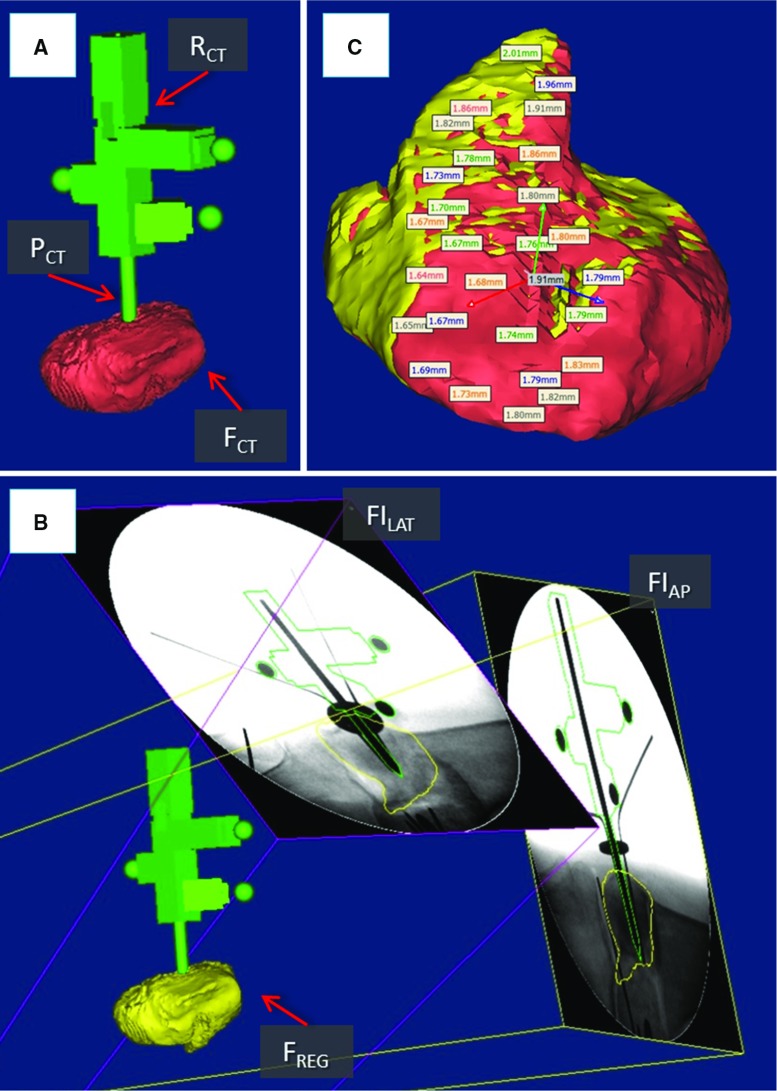
System evaluation. CT-generated 3D models of a fractured bone fragment $\hbox {F}_{{\mathrm{CT}}}$, orthopaedic pin $\hbox {P}_{{\mathrm{CT}}}$, and registration tool $\hbox {R}_{{\mathrm{CT}}}$ (**a**). Fluoroscopic images $\hbox {FI}_{{\mathrm{AP}}}$ and $\hbox {FI}_{{\mathrm{LAT}}}$ are registered with $\hbox {P}_{{\mathrm{CT}}}$ and $\hbox {R}_{{\mathrm{CT}}}$, and the 3D model of the bone fragment $\hbox {F}_{{\mathrm{REG}}}$ is registered with the fluoroscopic images (**b**). The registration accuracy is calculated as the difference between matching points on the fracture surfaces of $\hbox {F}_{{\mathrm{CT}}}$ (*red*) and $\hbox {F}_{{\mathrm{REG}}}$ (*yellow*) (**c**)

In a realistic surgical application, the orthopaedic pins should be inserted intra-operatively and in the operating theatre. However, with the objective of generating accurate ground truth registration, the orthopaedic pins were inserted into each bone fragment prior to the experiment. One orthopaedic pin was inserted into each bone fragment as described in the clinical workflow. One RT was placed on each pin, and CT scan data were acquired to generate 3D models of the broken bones (i.e. $\hbox {FEM}_{{\mathrm{CT}}}$, $\hbox {F1}_{{\mathrm{CT}}}$, $\hbox {F2}_{{\mathrm{CT}}})$, 3D models of the pins (i.e. $\hbox {P0}_{{\mathrm{CT}}}$, $\hbox {P1}_{{\mathrm{CT}}}$, $\hbox {P2}_{{\mathrm{CT}}})$, and 3D models of the RTs ($\hbox {RT0}_{{\mathrm{CT}}}$, $\hbox {RT1}_{{\mathrm{CT}}}$, $\hbox {RT2}_{{\mathrm{CT}}})$. We acquired two fluoroscopic images of each fragment–pin–RT at different angles (i.e. $\hbox {FI}_{{\mathrm{AP}}} = 90{^{\circ }}$, $\hbox {FI}_{{\mathrm{LAT}}} =60{^{\circ }})$—for a total of six images—which were imported in the reduction software together with the CT-generated 3D models of bones, RTs, and pins. Considering a generic fragment $\hbox {F}_{{\mathrm{CT}}}$, pin $\hbox {P}_{{\mathrm{CT}}}$, registration tool $\hbox {RT}_{{\mathrm{CT}}}$ (Fig. 9a), and two fluoroscopic images $\hbox {FI}_{{\mathrm{AP}}}$ and $\hbox {FI}_{{\mathrm{LAT}}}$, the proposed registration framework was applied as follows (Fig. 9b):

1. *Fluoroscopic image pose estimation:* The relative pose between $\hbox {FI}_{{\mathrm{AP}}}$ and $\hbox {FI}_{{\mathrm{LAT}}}$ with respect to $\hbox {RT}_{{\mathrm{CT}}}$ and $\hbox {P}_{{\mathrm{CT}}}$ was estimated as described in the previous section. However, in a real surgical application, $\hbox {RT}_{{\mathrm{CT}}}$ and $\hbox {P}_{{\mathrm{CT}}}$ are not available as the pins are inserted into the fragments *after* taking the CT scan. In this case, CAD models of the RT and pin, i.e. $\hbox {RT}_{{\mathrm{CAD}}}$ and $\hbox {P}_{{\mathrm{CAD}}}$, are used instead.
2. *Registration: *Once the relative pose of fluoroscopic images was established, the pose of the fractured bone fragment was estimated. The 3D model of the bone fragment $\hbox {F}_{{\mathrm{CT}}}$ was duplicated (Fig. 9b), generating an identical model $\hbox {F}_{{\mathrm{REG}}}$ which was registered with the fluoroscopic images as described in the previous section. $\hbox {F}_{{\mathrm{CT}}}$ was kept fixed and used as ground truth for the registration accuracy assessment.

To assess the registration framework, we used the following metrics: (1) surface target registration error (*sTRE*) [34] defined as the distance between matching points on the target bone model ($\hbox {F}_{{\mathrm{CT}}})$ and on the computed one ($\hbox {F}_{{\mathrm{REG}}}$, the pose has been established using the registration framework) (Fig. 9c). In this study, matching points to calculate *sTRE* were randomly selected on the *fracture surfaces* of the bone fragments. This is because the context of this study is image-guided robot-assisted fracture surgery which aims to accurately reduce a joint fracture by matching the fracture surfaces of the broken fragments. (2) Registration time.

**Table 1:** Results: assessment of the registration framework

Specimen$^{\mathrm{a}}$	sTRE (mm)			Registration time (min)
	FEM	F1	F2	
*#0 – Y,L* $^{\mathrm{b}}$	$1.16\pm 0.30$	$0.71\pm 0.17$	$0.77\pm 0.24$	26
*#1 – T,R*	$1.55\pm 0.97$	$1.15\pm 0.63$	$1.17\pm 0.43$	38
*#2 – Y,R*	$0.81\pm 0.16$	$1.22\pm 0.44$	$1.89\pm 0.24$	39
*#3 – T,L*	$0.76\pm 0.09$	$0.80\pm 0.19$	$1.40\pm 0.77$	52
*#4 – Y,L*	$0.71\pm 0.21$	$1.11\pm 0.22$	$1.37\pm 0.74$	40
*#5 – T,L*	$1.21\pm 0.23$	$1.33\pm 0.28$	$0.93\pm 0.34$	40
*#6 – T,R*	$0.73\pm 0.08$	$1.20\pm 0.45$	$1.17\pm 0.43$	38
*#7 – Y,L*	$0.88\pm 0.20$	$0.94\pm 0.54$	$1.07\pm 0.35$	35
*#8 – Y,L*	$1.01\pm 0.22$	$1.13\pm 0.23$	$1.10\pm 0.48$	38
*#9 – Y,R*	$0.91\pm 0.21$	$1.12\pm 0.52$	$1.08\pm 0.55$	35
*#10 – Y,L*	$1.08\pm 0.54$	$0.77\pm 0.15$	$1.26\pm 0.75$	36
*#11 – Y,R*	$1.11\pm 0.42$	$1.29\pm 0.72$	$0.94\pm 0.25$	38
*#12 – T,L*	$1.12\pm 0.30$	$2.62\pm 1.07$	$1.35\pm 0.35$	35
*#13 – T,L*	$1.24\pm 0.58$	$1.53\pm 0.65$	$1.33\pm 0.58$	32

$^{\mathrm{a}}$ $\hbox {T} =$ T-shaped 33-C1 fracture; $\hbox {Y} = \hbox {Y-shaped}$ 33-C1 fracture; $\hbox {R} = \hbox {right limb}$; $\hbox {L} = \hbox {left limb}$

$$^{\mathrm{b}}$$ Plastic model

### Laboratory test on plastic models

A left lower limb model was manufactured by Sawbones (Vashon Island, WA, USA) including a Y-shaped 33-C1 distal femur fracture, tibia, and simulated knee ligaments, i.e. ACL, PCL, LCL, and MCL, made of polypropylene bands [35]. The goal of this experiment was to prove the applicability of the proposed registration framework and evaluate its accuracy on a simple model, i.e. excluding muscles and flesh. The protocol described in the “Evaluation methodology” section was applied to each fragment. Results are reported in Table 1.

### Cadaveric trials

Trials on cadavers were conducted to assess the reduction framework applicability and accuracy in a realistic surgical environment. Thirteen lower limb fresh cadaveric specimens were fractured by a surgeon to generate T- and Y-shaped 33-C1 distal femur fractures, namely 6 T-shaped and 7 Y-shaped fractures on both left and right limbs. The reduction framework was applied to each fragment of each specimen as described above. Results are reported in Table 1.

Moreover, 4 fractures (2 T-shaped and 2 Y-shaped) were physically reduced using the RAFS surgical system following the new clinical workflow. The reduction accuracy, defined as the restoration of the normal anatomical alignment, was assessed by a surgeon by measuring the residual translations and angulations at different points on fluoroscopic images taken once the reduction was completed [36]. The reduction accuracy is expressed as translational and rotational root-mean-squared error (RMSE) [7]. Results are reported in Table 2.

**Table 2:** Results: fracture reduction accuracy using RAFS system

Specimen$^{\mathrm{a}}$	Reduction accuracy—RMSE$^{\mathrm{b}}$ $\Delta \hbox {T} (\hbox {mm})$, $\Delta \hbox {R}({^{\circ }})$		Surgery time (min)
	F1	F2	
*#1 – T,R*	$1.41\pm 0.30\,\hbox {mm}$	$0.93\pm 0.20\,\hbox {mm}$	106
	$3.12\pm 0.40{^{\circ }}$	$3.30\pm 0.50{^{\circ }}$	
*#2 – Y,R*	$0.85\pm 0.30\,\hbox {mm}$	$1.83\pm 0.10\,\hbox {mm}$	118
	$2.20\pm 0.10{^{\circ }}$	$2.40\pm 0.30{^{\circ }}$	
*#3 – T,L*	$1.00\pm 0.40\,\hbox {mm}$	$1.38\pm 0.40\,\hbox {mm}$	115
	$2.40\pm 0.20{^{\circ }}$	$2.40\pm 0.60{^{\circ }}$	
*#7 – Y,L*	$1.04\pm 0.25\,\hbox {mm}$	$1.13\pm 0.01\,\hbox {mm}$	112
	$0.12\pm 0.05{^{\circ }}$	$0.69\pm 0.04{^{\circ }}$	

$^{\mathrm{a}}$ $\hbox {T} =$ T-shaped 33-C1 fracture; $\hbox {Y} = \hbox {Y-shaped}$ 33-C1 fracture; $\hbox {R} = \hbox {right limb}$; $\hbox {L} = \hbox {left limb}$

$^{\mathrm{b}}$ Translational error $\Delta \hbox {T}$ calculated over 12 data points; rotational error $\Delta \hbox {R}$ calculated over 2 data points

**Fig. 10:**
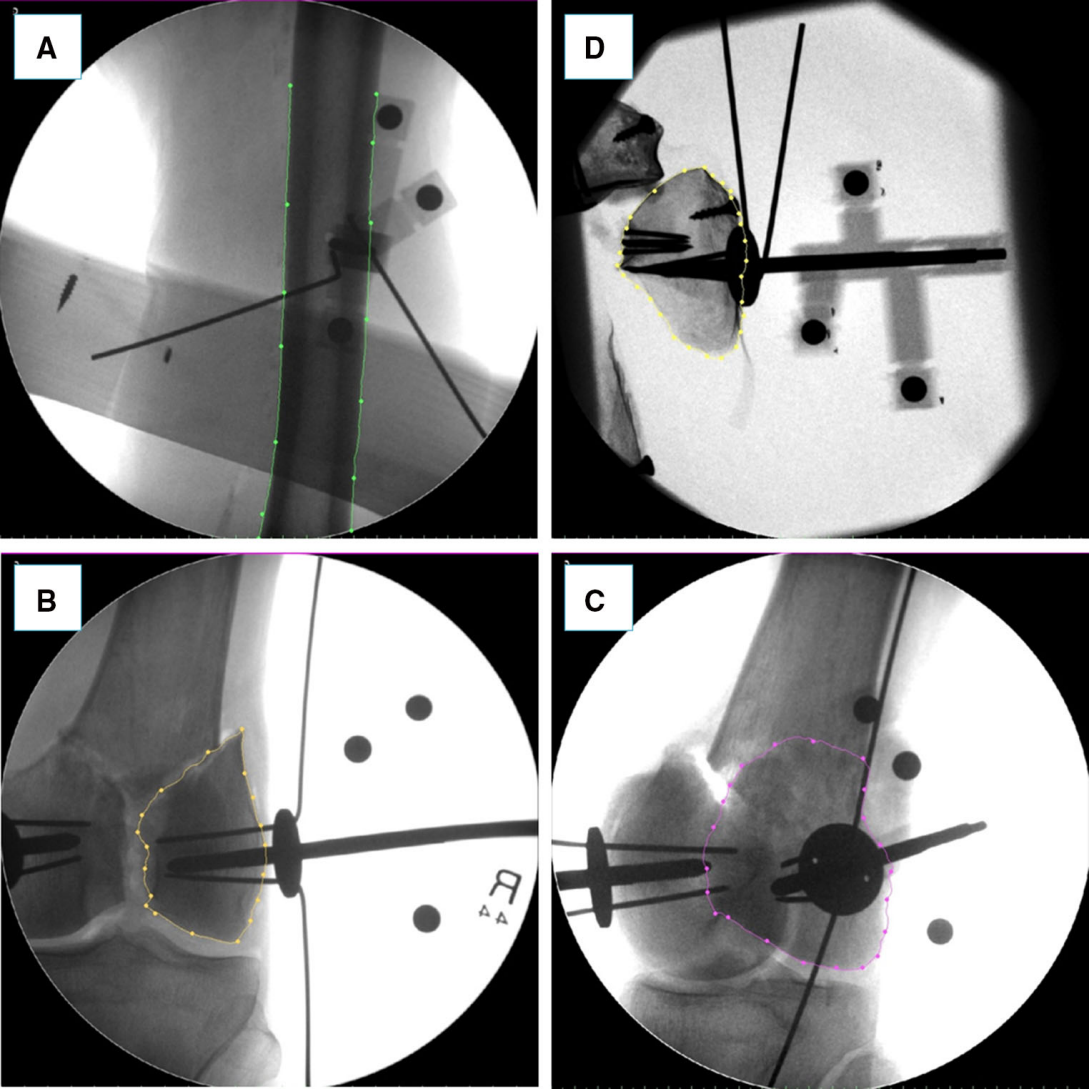
Example of fluoroscopic images from validation experiments on cadavers (**a**, **b**, **c**) and plastic phantom (**d**). A cadaveric specimen’s femur is segmented (green contour) in the frontal plane (**a**). A fractured bone fragment is segmented in the frontal (*yellow contour* in **b**) and lateral (*purple contour* in **c**) planes: the segmentation in the lateral plane is more challenging as the two broken fragments are overlapped. The segmentation of the broken fragments in the plastic phantom is easier as the soft tissue is not present (**d**)

## Discussion

In this paper, we have introduced a registration framework enabling intra-operative image guidance for the RAFS surgical system. We have evaluated the registration framework in a simple laboratory test on bone phantoms and through cadaveric trials to assess its accuracy and applicability to a realistic surgical procedure. Experimental results demonstrated the potential of the proposed registration framework for improving the feasibility of the RAFS system.

The experimental results on the bone phantoms showed registration accuracies (*sTRE*) of $1.16\pm 0.30\,\hbox {mm}$ (FEM), $0.71\pm 0.17\,\hbox {mm}$ (F1), and $0.77\pm 0.24\,\hbox {mm}$ (F2). Cadaveric trials resulted in average registration accuracy of $1.01\pm 0.65\,\hbox {mm}$ (FEM), $1.25\pm 0.60\,\hbox {mm}$ (F1), and $1.16\pm 0.64\,\hbox {mm}$ (F2). These results demonstrated better performance of the proposed framework in terms of accuracy with respect to other studies reported in the literature [34, 37]. A comparison of the laboratory and cadaveric trials results shows that the registration accuracy of the femur (FEM) (Fig. 10a) is quite similar in both cases, while the registration accuracy of fragments F1 and F2 is almost 40% lower (higher *sTRE*) in the cadaveric trials. This is mostly due to the higher difficulty of segmenting the contour of fractured fragments of the distal portion of the femur on the fluoroscopic images because of: (1) the presence of complex soft tissue structures encapsulating the fragments (i.e. ligaments, tendons, muscles, flesh) (e.g. compare Fig. 10b, d); (2) the fragments overlapped in the fluoroscopic image taken from the lateral view (Fig. 10c). In particular, the segmentation of the distal fragments in specimens #12 and #13 required extensive manual intervention, resulting in the worst registration accuracies measured in the experiments. The segmentation of the femur shaft was much easier to achieve and did not require any manual intervention.

The proposed registration framework was successfully integrated into the navigation system, and four reductions were achieved using the RAFS system on four cadaveric specimens following the new clinical workflow. The RAFS system showed reduction accuracies that can be considered clinically acceptable (Table 2) [7] on both Y- and T-shaped fractures, and left and right lower limbs. Average residual reduction errors (RMSE) of only $1.2\pm 0.3\,\hbox {mm}$ and $2\pm 1{^{\circ }}$ were achieved, confirming (see [7]) the higher accuracy of the RAFS system with respect to other systems for fracture surgery reported in the literature [10–16].

Cadaveric trials showed that the average surgical time to reduce the fractures using the proposed registration framework and the RAFS system was about 113 min, including pins insertion, image registration framework, robot set-up, and fracture reduction (Table 2). The image registration required on average almost 40 min to be completed, as reported in Table 1. Although the registration framework resulted quite accurate and allowed the RAFS system to be used in the surgical scenario, it proved to be time-consuming at this stage of development. This is mainly due to the manual user interaction required to (1) identify the location of feature geometries of the RT (beads) and the pin in the fluoroscopic images and (2) segment the fragment contour on the fluoroscopic images to achieve the registration. Automating the registration framework could minimise (possibly avoid) the extended manual interaction and reduce the registration time to only a few seconds. This can be achieved through image processing. The identification of the RT (beads) and pin (sharp end) features in the fluoroscopic images (which is currently done manually by the user) could be automated using a pattern-matching algorithm searching for the desired features [38, 39]. Regarding the registration of the bone model with the fluoroscopic images, one of the main issues encountered, as mentioned before, was the overlap of the bone fragments in the fluoroscopic image taken from the lateral view. Therefore, the segmentation of the bone fragments in the lateral fluoroscopic image required an extensive manual intervention. One solution could be pose optimisation of the fluoroscope in order to obtain fluoroscopic images with no, or minimum, overlap of the fragments which can potentially be segmented automatically. This method, proposed for angiography interventions [40], could be used for fracture surgeries. Also, the image processing algorithms (i.e. segmentation and registration algorithms) can be optimised and implemented on a graphics processing unit (GPU) as proposed in [37], considerably reducing the whole processing time and, consequently, the registration time. Therefore, the whole surgical procedure would benefit from this, as the surgery time could be potentially reduced to about 1 h. Moreover, the surgical procedure would be even safer, as one potential source of error during the fracture reduction using the robotic system is the displacement of the orthopaedic pins with respect to the bone fragments due to the high forces involved, e.g. the pins bend and/or rotate inside the bones. The manipulation pin has been designed to avoid these issues by attaching an anchoring system that prevents the pin to rotate inside the bone. This can support loads of up to 150N/7 Nm with minimum bending (<0.5 mm) [22, 23]. However, if the anchoring system and/or the pin should fail, then the consequent displacement would result in the loss of the image guidance as the relative pose on the pin with respect to the bone would be lost, and the registration procedure would have to be repeated. A fast registration framework would allow a quick recovery of the image guidance.

Future work will explore the automation of the registration framework, to make the whole surgical procedure quicker and more precise. Also, although the proposed framework was designed for the surgical treatment of distal femur fractures using the RAFS system, we believe that the developed technology can be successfully applied to other different surgical applications where image guidance is required, such as hip, spinal, and/or upper limb surgery. This would require an optimisation and redesign of the registration tool and the orthopaedic manipulation pin.

## Conclusion

This paper presented an image registration framework enabling image guidance for the RAFS surgical system. The proposed registration framework along with the new clinical workflow allows the intra-operative registration of pre-operative CT images and intra-operative fluoroscopic images using a custom-made fiducial, improving the earlier version of the navigation system. Experimental trials demonstrated that the registration framework is reliable and effective and can achieve intra-operative registration with high level of accuracy. Fracture reduction trials on cadaveric specimens demonstrated that the registration framework and the new clinical workflow allow the reduction of complex knee fractures using the RAFS system in a realistic surgical scenario. At this stage of development, the registration framework requires manual user interaction and extended the registration time. Automating the registration is the focus of our future research.
